# Treatment response amplitude and timing in chronic inflammatory demyelinating polyneuropathy with routine care: Study of a UK cohort

**DOI:** 10.1111/ene.16399

**Published:** 2024-07-09

**Authors:** Yusuf A. Rajabally, Young Gi Min, Kabir K. Nazeer, Christina Englezou

**Affiliations:** ^1^ Aston Medical School, Aston University Birmingham UK; ^2^ Department of Neurology Inflammatory Neuropathy Clinic, University Hospitals Birmingham Birmingham UK; ^3^ Department of Translational Medicine Seoul National University College of Medicine Seoul South‐Korea

**Keywords:** chronic inflammatory demyelinating polyneuropathy, outcome, response, timing, treatment

## Abstract

**Background and purpose:**

The amplitude, timing, and determinants of improvement with available treatments are uncertain in chronic inflammatory demyelinating polyneuropathy (CIDP). Our primary objective was to quantify categorized outcomes with routine care.

**Methods:**

We retrospectively studied treatment response within 36 months from initiation in 112 consecutive subjects with CIDP. Response was classified into a proposed new "CIDP treatment‐response category" (CT‐RC), based on achieved endpoints. Determinants of the CT‐RC, of timing of maximum improvement, and of treatment discontinuation were ascertained.

**Results:**

The CT‐RC demonstrated high concurrent validity with current outcome measures. Thirty‐six subjects (32.1%) achieved a “complete response,” 37 (33%) a “good partial response,” 10 (8.9%) a “moderate partial response,” and 15 (13.4%) a “poor partial response.” Fourteen subjects (12.5%) were “nonresponsive.” The CT‐RC was independently predicted only by age. Mean time to maximum improvement was 12.1 months (range = 1–36) and was not associated with any pretreatment covariate. Treatment discontinuation occurred in 24 of 62 (38.2%) partial responders and was only associated with shorter pretreatment disease duration. Nonresponders were older and received a similar number of treatments compared to responders.

**Conclusions:**

CT‐RC classification indicates persistent disability in >60% of treatment responders in CIDP. Timing of maximum improvement is variable, frequently delayed, and unpredictable. Treatment withdrawal without deterioration is achievable in approximately 40% of subjects and may be more likely with prompt treatment. Treatment withdrawal in partial responders and limited escalation in nonresponders suggest implication of physician‐ and patient‐related factors in suboptimal response. More effective treatments/treatment methods and better understanding of other factors influencing response are needed in CIDP.

## INTRODUCTION

Chronic inflammatory demyelinating polyneuropathy (CIDP) is the commonest treatable chronic autoimmune neuropathy [1]. Improvement of varying degrees, normalization, remission, and refractoriness are all described [2], although proportions of well‐defined outcomes with routine care remain unknown.

Randomized controlled trials have used as primary outcome measure improvement by a standardized margin considered to represent the minimum clinically important difference (MID) for the scale [3]. The most commonly utilized scale has been the adjusted Inflammatory Neuropathy Cause and Treatment (INCAT) [4], with a degree of improvement of 1 point [4, 5, 6, 7, 8]. The reliance on this degree of response is, however, considered to be frequently inadequate in practice, mainly because it has vastly different implications depending on pretreatment disability and individual patient characteristics.

The time frame used to assess treatment response is equally very variable, as are the conditions justifying treatment reduction or withdrawal. Although the latest European Academy of Neurology/Peripheral Nerve Society (EAN/PNS) 2021 guidelines [9] have been helpful to summarize evidence and management modalities, there is a paucity of data on treatment outcomes with routine care.

New drugs have been and still are under investigation for CIDP [10]. Even if enlarging the therapeutic armamentarium is highly desirable, lack of data on treatment response with currently available agents makes identifying the place of new agents a difficult task.

Our primary objective was to study the amplitude of the maximum response and its timing in a treated cohort of subjects with CIDP within a prespecified time frame. We proposed to (i) meaningfully categorize response, (ii) establish in what proportions these response levels were achieved, and (iii) determine the time taken to reach these levels. We also aimed to ascertain the rate of treatment cessation and its determinants and to identify potential causes of suboptimal outcomes in routine care.

## METHODS

### Study subjects

We performed a retrospective review of electronic records of all consecutive patients presenting with a clinical diagnosis of CIDP, of all subtypes, meeting EAN/PNS 2021 guidelines [9] for a diagnosis of “CIDP,” having received treatment at the Inflammatory Neuropathy Service, University Hospitals Birmingham, UK. The study period was between July 2014 and January 2024. We selected patients who had received their first‐ever treatment for CIDP, as well as those who were started on any new treatment or treatment regimen, as per our protocols, after first assessment at our centre, and had subsequently been followed up for at least 36 months or achieved full normalization of function at any time within 36 months.

### Data collection

We determined (i) demographics, (ii) CIDP disease subtype, (iii) disease duration from onset to time of treatment initiation at our centre, (iv) acuteness of presentation, and (v) presence of comorbidities with functional impact, at first evaluation in our service. For the purposes of this study, we considered exclusively the Overall Neuropathy Limitation Score (ONLS) [11]. The ONLS scale is systematically utilized at clinic attendances in our practice (5 points for upper limb score, 7 points for lower limb score; optimal score = 0). The ONLS, derived from the INCAT scale [4], is preferred in our practice because of its ability to distinguish levels of mobility impairment in greater detail than the INCAT. Best achieved concurrent posttreatment Medical Research Council sum score (MRCSS) and Inflammatory Rasch‐Built Overall Disability Scale (I‐RODS) [12] were collected.

We also aimed to determine, secondarily, associations of pretreatment electrophysiological measures with response. We recorded pretreatment summated compound muscle action potential values (ƩCMAP), adding the distal CMAP evoked for unilateral median/ulnar/common peroneal/tibial nerves, and summated sensory nerve action potential values (ƩSNAP), adding unilateral sural and radial SNAPs.

### Treatment protocols

**FIGURE 1:**
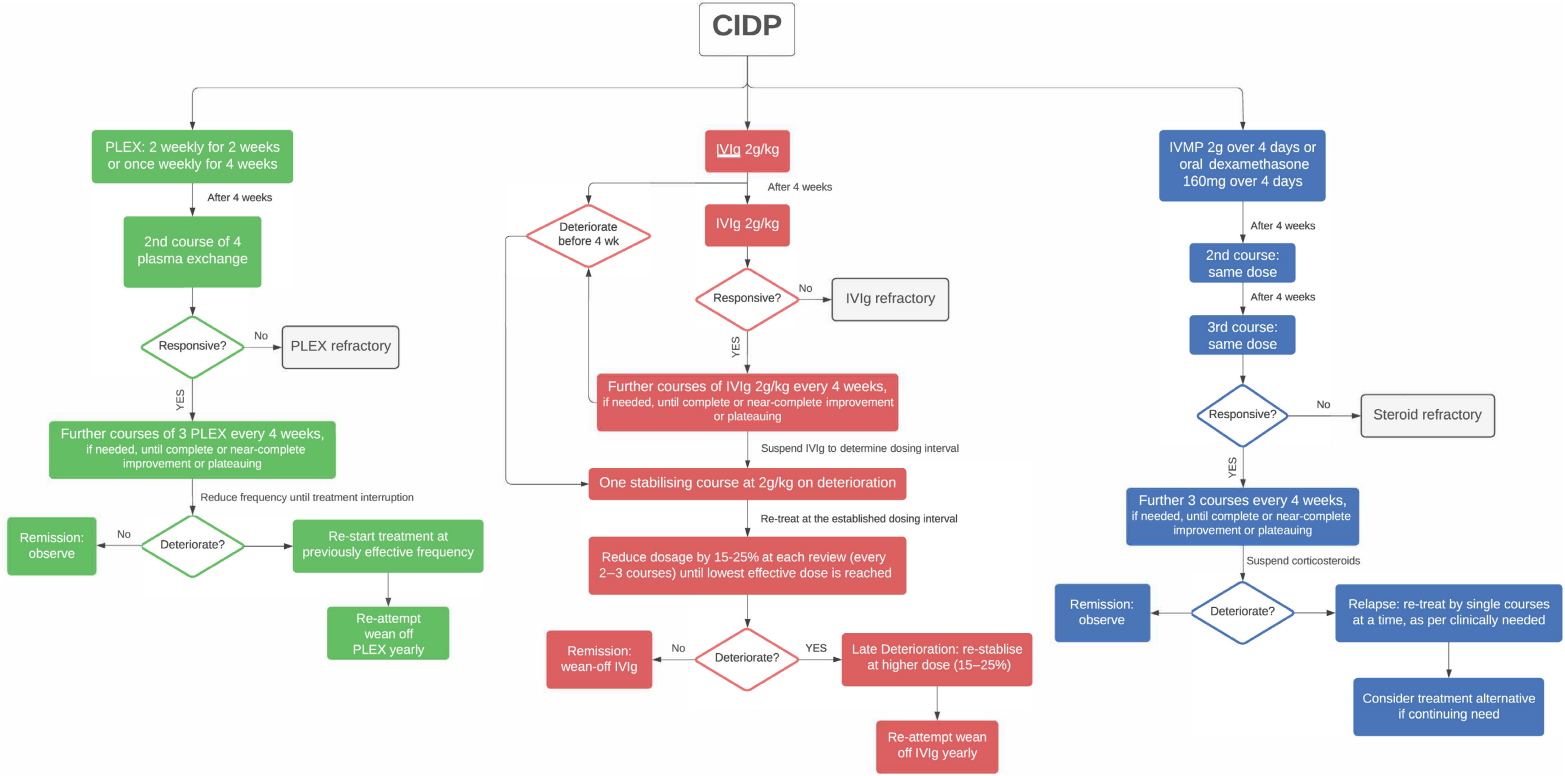
First‐line treatment protocols with immunoglobulins, corticosteroids, and plasma exchange (PLEX) used in 112 consecutive subjects with chronic inflammatory demyelinating polyneuropathy (CIDP). IVIg, intravenous immunoglobulins; IVMP, intravenous methylprednisolone.

First‐line therapeutic protocols used in our unit are summarized in Figure 1. In newly diagnosed subjects and those with relapsing disease but previously in remission off treatment, one first‐line treatment was attempted. Intravenous immunoglobulin therapy, or alternatively, pulse intravenous methylprednisolone was commenced as per established protocols, based on existing evidence [7, 13, 14]. In the presence of contraindications or immunoglobulin supply issues, as during the first months of the SARS‐CoV‐2 pandemic in 2020, plasma exchanges were used as first treatment [15]. Switching of first‐line therapy was done in the absence of response, as established in our practice, and as described elsewhere [16]. Eventual subsequent steps in the case of unresponsiveness included use of a third first‐line agent alone, combination first‐line therapy, and in cases where these measures were unhelpful, immunosuppression through one of two agents: rituximab (2 g intravenously over 2 weeks, and 1 g 6 months later in incomplete responders) or cyclophosphamide (1 g/m^2^, administered intravenously, monthly for 6 months) [17]. For previously diagnosed and treated patients referred to our service, treatment modifications were performed in situations of persistent disability, due to lack/absence of treatment effect. These included dose increase and frequency increase, followed by other aforementioned subsequent steps, as offered to newly diagnosed patients.

The timing of therapeutic initiation and baseline pretreatment disability levels were defined as the time of the first treatment in our unit. We considered a time frame of a maximum of 36 months for evaluation of treatment effects. The maximum treatment effect attained (best total ONLS) and maintained for at least 3 months was ascertained for all subjects followed up for ≥36 months at our centre, during the 36 months following treatment initiation. For those having a follow‐up period of ≤36 months, only the subjects having attained a total ONLS score of 0, or 1 (from the upper limb subscore only), maintained for at least 3 months were included.

### Definition of response score and CIDP treatment‐response category

**FIGURE 2:**
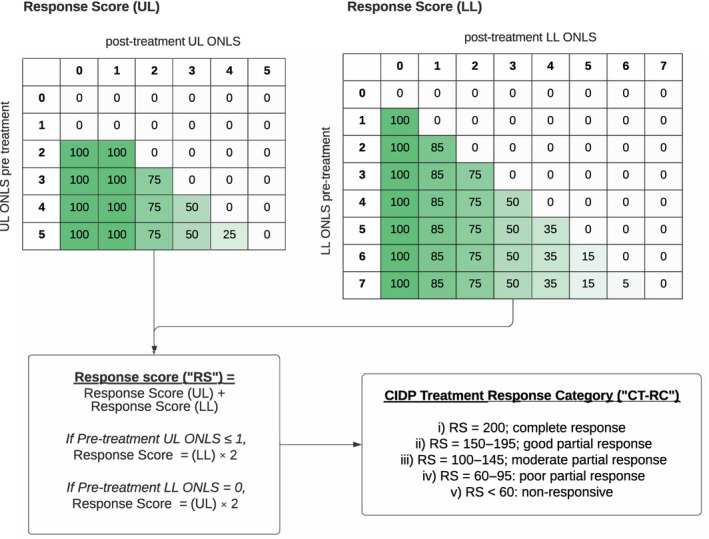
Classification algorithm for the chronic inflammatory demyelinating polyneuropathy (CIDP) treatment‐response category using the response score from score improvements in upper limb (UL) and lower limb (LL) components of the Overall Neuropathy Limitation Score (ONLS) in 112 subjects with treated CIDP.

We considered, as an underlying principle, that complete treatment response corresponds to normalization of function. We concluded that response should therefore not depend on pretreatment status nor on amplitude of improvement. To generate an exclusively result‐based categorical classification of response, we used a point allocation system (of 0–100) based on posttreatment ONLS disability levels in the upper limb (UL) and lower limb (LL) subscores (Figure 2). The point allocation was arbitrarily established on the estimated percentage residual disability, corresponding to each ONLS subscore, in relation to the level above. The UL and LL subscores were added, producing a “response score” (RS). In the absence of improvable disability in either subscore (pretreatment UL ONLS ≤ 1 or pretreatment LL ONLS = 0), the point allocation for the affected pretreatment subscore was doubled. The RS was then assigned to a “CIDP treatment‐response category” (CT‐RC), based on posttreatment disability level, as follows: (i) CT‐RC 1 = 200 points, “complete response” (corresponding to full recovery); (ii) CT‐RC 2 = 150–195 points, “good partial response” (equivalent to at least having the ability to do all common self‐care tasks and ability to walk without aid); (iii) CT‐RC 3 = 100–145 points, “moderate partial response” (equivalent to at least having the ability to do most but not all common self‐care tasks and ability to walk with unilateral support); (iv) CT‐RC 4 = 60–95 points, “poor partial response” (equivalent to at least having purposeful UL movements without the ability to perform any common self‐care task and ability to walk with bilateral support); and (v) CT‐RC 5 = <60 points, “non‐responsive” (corresponding to no or no meaningful change from pretreatment level).

### Statistical analysis

CT‐RC frequency distribution was established. CT‐RC concurrent validity was evaluated by comparison with best achieved posttreatment MRCSS and I‐RODS, using Spearman rank correlation tests. Determinant(s) of the CT‐RC were ascertained. Timing of maximum improvement achieved and maintained for 3 months and its determinants were established. Proportions of subjects in each CT‐RC taken off treatment were established, and predictors of treatment discontinuation were determined. The determinants of ONLS improvement were ascertained.

Statistical analyses were performed with SPSS 28.0 (IBM, Armonk, NY, USA) and R software (v4.2.1). Comparison of proportions were performed by Fisher exact tests and comparison of means by independent *t*‐tests or analysis of variance as appropriate. Correlations were performed by Spearman rank correlation tests. Bonferroni corrections were applied for multiple testing. Independent associations were sought through linear regression, considering in the models the relevant covariates demonstrating association on Spearman rank correlation after Bonferroni correction. Significance was set at *p* < 0.05, for all tests, before Bonferroni corrections.

### Approvals

This study was reviewed and approved as part of a retrospective clinical audit of treatment outcomes in patients with CIDP attending our service (CARMS‐20702, 23 October 2023). Clinical audit does not require ethics committee approval in the UK.

## RESULTS

### Baseline characteristics

From a cohort of 214 consecutive subjects registered at our service with a diagnosis of “suspected CIDP,” we identified 112 subjects meeting the inclusion criteria for the current study.

The main characteristics of recruited subjects are summarized in Table 1. There were 39 females and 73 males (ratio = 1:1.9). Mean age was 62.5 years (SD = 14.5), and mean disease duration before treatment initiation at our centre was 40.4 months (SD = 59.1). Seventy subjects (62.5%) were newly diagnosed with CIDP, and 42 (37.5%) had been referred after unsuccessful or unsatisfactory treatment for CIDP commenced at other institutions. Eighty‐seven subjects (77.7%) had typical CIDP, 17 (15.2%) had variant multifocal CIDP, four (3.6%) had motor CIDP, and four (3.6%) had sensory CIDP. Twenty‐two (19.6%) presented with acute‐onset CIDP. Twenty‐seven (24.1%) had an associated neurological/rheumatological/cardiorespiratory comorbidity impacting on physical function.

**TABLE 1:** Characteristics of 112 consecutive subjects with CIDP treated with routine care and followed up for 36 months or having attained full normalization of function before 36 months.

Characteristic	Value
Mean age, years (SD)	62.5 (14.5)
Gender, F:M	39:73 (1:1.9)
Mean disease duration before treatment initiation, months (SD)	40.4 (59.1)
CIDP subtype proportions	Typical CIDP: 87/112 (77.7%)
	Variant multifocal CIDP: 17/112 (15.2%)
	Motor CIDP: 4/112 (3.6%)
	Sensory CIDP: 4/112 (3.6%)
Proportion with acute onset CIDP	22/112 (19.6%)
Rate of associated comorbidity (neurological, rheumatological, cardiorespiratory) impacting on mobility	27/112 (24.1%)
Mean pretreatment total ONLS (SD)	5.35 (2.71)
Mean posttreatment total ONLS (SD)	2.12 (2.02)
Mean total ONLS improvement (SD)	3.23 (2.74)

Abbreviations: CIDP, chronic inflammatory demyelinating polyneuropathy; F, female; M, male; ONLS, Overall Neuropathy Limitation Score.

Mean pretreatment total ONLS was 5.35 (range = 2–12, SD = 2.71), mean pretreatment UL ONLS was 2.29 (range = 0–5, SD = 1.22), and mean pretreatment LL ONLS was 3.02 (range = 0–7, SD = 1.95). Mean posttreatment total ONLS was 2.12 (range = 0–10, SD = 2.02), mean posttreatment UL ONLS was 0.81 (range = 0–4, SD = 1.0), and mean posttreatment LL ONLS was 1.36 (range = 0–6, SD = 1.33). The mean total ONLS improvement with treatment was 3.23 (range = 3–12, SD = 2.74, *p* < 0.001).

Concurrent best achieved MRCSS (mean = 74.8/80, SD = 7.7) was available for 103 subjects and raw I‐RODS (mean = 36.3/48, SD = 10.3) for 84 subjects.

### Frequency distribution of CT‐RC

**FIGURE 3:**
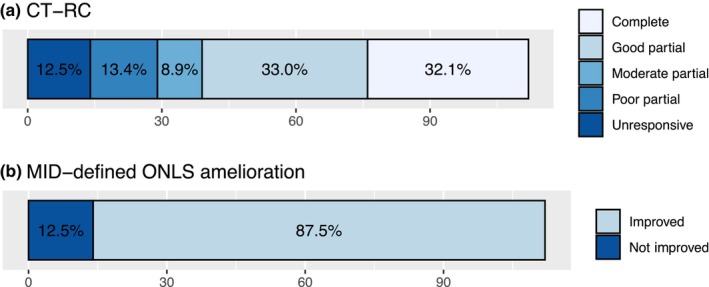
Value of chronic inflammatory demyelinating polyneuropathy (CIDP) treatment‐response category (CT‐RC) versus minimum clinically important difference (MID)‐defined Overall Neuropathy Limitation Score improvement in evaluating residual disease burden with routine care in a cohort of 112 consecutive subjects with CIDP.

The results of treatment‐response category classification and ongoing treatment status at the time of analysis are shown in Table 2 and Figure 3. The median RS was 175 (range = 0–200, interquartile range = 113.75). Through grouping into the CT‐RC, 36 subjects (32.1%) were classified in CT‐RC 1 (“complete response”), 37 (33%) in CT‐RC 2 (“good partial response”), 10 (8.9%) in CT‐RC 3 (“moderate partial response”), and 15 (13.4%) in CT‐RC 4 (“poor partial response”). Fourteen subjects (12.5%) were classified in CT‐RC 5 (“non‐responsive”), for whom ONLS scores were unchanged in 12 and worse in two.

**TABLE 2:** CT‐RC distribution and treatment status at time of study in 112 consecutive subjects with CIDP treated with routine care.

CT‐RC	Proportion (%)	Proportion of treatment cessations at time of study (%)
CT‐RC 1: complete response	36/112 (32.1%)	19/36 (52.8%)
CT‐RC 2: good partial response	37/112 (33%)	16/37 (43.2%)
CT‐RC 3: moderate partial response	10/112 (8.9%)	3/10 (30%)
CT‐RC 4: poor partial response	15/112 (13.4%)	5/15 (33%)
CT‐RC 5: nonresponsive	14/112 (12.5%)	4/14 (28.6%)^a^

Abbreviations: CIDP, chronic inflammatory demyelinating polyneuropathy; CT‐RC, CIDP treatment‐response category.

^a^ Excluded from further analysis.

In the 98 responders, the effective treatment was immunoglobulins in 64 (65.3%), corticosteroids in 11 (11.2%), and plasma exchange in 10 (10.2%). Combination of two first‐line treatments was required in nine (9.2%), rituximab in three (3.1%), and cyclophosphamide in one (1%).

### Concurrent validity of CT‐RC with existing outcome measures

The CT‐RC correlated highly with post‐treatment ONLS (Spearman *ρ* = 0.88, *p* < 0.001), posttreatment MRCSS (Spearman *ρ* = −0.73, *p* < 0.001), and post‐treatment I‐RODS (Spearman *ρ* = −0.72, *p* < 0.001).

### Time to maximum improvement in responders

**FIGURE 4:**
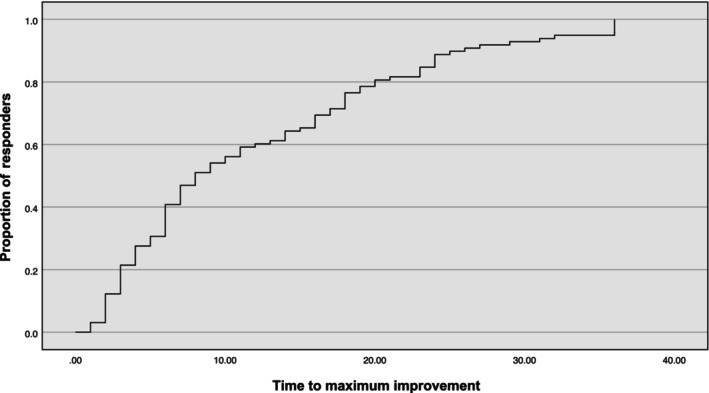
Proportion of subjects achieving maximum response within a 36‐month follow‐up period in 112 consecutive subjects with chronic inflammatory demyelinating polyneuropathy (CIDP) receiving routine care. CT‐RC, CIDP treatment‐response category; MID, minimum clinically important difference; ONLS, Overall Neuropathy Limitation Score.

Proportions of subjects attaining maximum improvement with time are shown in Figure 4. Mean time to maximum ONLS improvement was 12.1 months (range = 1–36, SD = 9.7). Among the 98 responders, 21 (21.4%) reached their maximum ONLS improvement within 3 months of treatment initiation, 40 (40.8%) within 6 months, and 59 (60.2%) within 12 months. Twenty‐eight (28.6%) demonstrated their maximum ONLS improvement during the second year of treatment and 11 (11.2%) during the third year of treatment. No plateauing of the proportion of subjects attaining maximum improvement was observed during the 36‐month follow‐up period (Figure 4). There was no significant difference in time to maximum improvement (*p* = 0.13) nor in proportion of subjects with delayed maximum improvement at >12 months (*p* = 0.14), comparing complete (CT‐RC 1) and partial responders (CT‐RC 2–4). Responders having received a single treatment had a shorter mean time to maximum improvement than those having received multiple treatments (9.5 vs. 16.0 months, *p* = 0.002) but also had lower pre‐treatment total ONLS (mean = 4.5 vs. 6.6, *p* < 0.001).

### Rate of treatment cessation versus continuation

Of the 36 subjects in CT‐RC 1, 19 (51.4%) were in remission off treatment, withdrawn without ONLS deterioration for at least 3 months, whereas 17 (48.6%) remained on treatment with confirmed dependency through unsuccessful dose reduction/withdrawal trials. Proportions of subjects having undergone treatment withdrawal versus subjects remaining on continuing treatment were 43.2% versus 56.8%, 30% versus 70%, and 33% versus 67%, for CT‐RC 2, CT‐RC 3, and CT‐RC 4, respectively (Table 2). Although excluded from further analysis, 10 of 14 subjects (71.4%) in CT‐RC 5 (non‐responders) remained on continuing treatment, as a result of improvement on other scales, exceeding the MID, without concurrent total ONLS change. Twenty‐four of 62 (38.7%) partial responders (CT‐RC 2–4) were hence off treatment with stable ONLS for a least 3 months. There was no difference between the partial responders taken off treatment and those on continuing treatment with regard to age (*p* = 0.1), presence of comorbidity (*p* = 0.36), total pretreatment ONLS (*p* = 0.08), or time to maximum improvement (*p* = 0.13). Disease duration was shorter (mean = 12.0 vs. 46.8 months, *p* = 0.006), total ONLS improvement was larger (mean = 4.25 vs. 2.87, *p* = 0.04), and typical CIDP was more frequent (23/24 vs. 28/38, *p* = 0.04) in partial responders having undergone treatment withdrawal than in those on continuing treatment.

### Comparison between responders and non‐responders

Responders (CT‐RC 1–4) were younger than non‐responders (CT‐RC 5; mean = 60.8 vs. 73.1 years, *p* = 0.002) but had comparable disease durations (mean = 37.2 vs. 67.1 months, *p* = 0.08), associated comorbidity (6/14 vs. 21/98, *p* = 0.1), and pre‐treatment total ONLS scores (5.37 vs. 5.14, *p* = 0.77). Mean number of different treatments used was not significantly different in responders versus non‐responders (1.57 vs. 2.00, *p* = 0.07).

### Associations of CT‐RC, treatment withdrawal, time to maximum improvement, and total ONLS improvement

**FIGURE 5:**
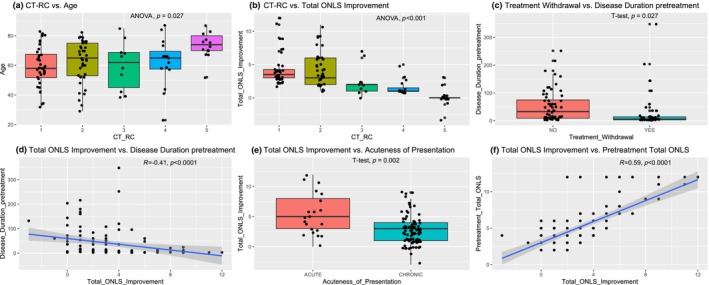
Boxplot/graphical representations of significant associations found in 112 consecutive subjects with chronic inflammatory demyelinating polyneuropathy (CIDP) treated with routine care between (a) CIDP treatment‐response category (CT‐RC) and age, (b) CT‐RC and total Overall Neuropathy Limitation Score (ONLS) improvement, (c) treatment withdrawal and disease duration pretreatment, (d) total ONLS improvement and disease duration pretreatment, (e) total ONLS improvement and acuteness of presentation, and (f) total ONLS improvement and total pretreatment ONLS. ANOVA, analysis of variance.

The findings of Spearman rank correlation tests with main relevant covariates are shown in Table 3 and Figure 5. The CT‐RC correlated with age (*p* = 0.005) and inversely with ONLS improvement (*p* < 0.001) but not with pre‐treatment ONLS. There was no association of the CT‐RC with disease duration before treatment initiation, CIDP subtype, presence of comorbidities, or acuteness of presentation. Time to maximum improvement did not correlate with any pretreatment covariate. Treatment discontinuation correlated only inversely with disease duration before treatment initiation (*p* < 0.001). Amplitude of ONLS improvement correlated inversely with disease duration before treatment initiation (*p* < 0.001), directly with acuteness of presentation (*p* < 0.001), and with pre‐treatment total ONLS (*p* < 0.001).

**TABLE 3:** Significance levels of Spearman rank correlations (after Bonferroni correction for the seven studied covariates [corrected *p*‐value significance level: *p* < 0.007]) for CT‐RC, treatment withdrawal at time of study, time to maximum improvement, and total ONLS improvement, with relevant covariables, in the total cohort of 112 consecutive subjects with treated CIDP.

	Age	Disease duration before treatment initiation	Typical CIDP subtype	Acuteness of presentation	Presence of comorbidity	Pretreatment total ONLS	Total ONLS improvement
CT‐RC	*ρ*: 0.262, *p* = 0.005	NS	NS	NS	NS	NS	*ρ*: −0.592, *p* < 0.001^a^
Time to maximum improvement	NS	NS	NS	NS	NS	NS	NS
Treatment withdrawal	NS	*ρ*: 0.390, *p* < 0.001^a^	NS	NS	NS	NS	NS
Total ONLS improvement	NS	*ρ*: −0.414, *p* < 0.001^a^	NS	*ρ*: 0.332, *p* < 0.001	NS	*ρ*: 0.593, *p* < 0.001	–

Abbreviations: CIDP, chronic inflammatory demyelinating polyneuropathy; CT‐RC, CIDP treatment‐response category; NS, nonsignificant; ONLS, Overall Neuropathy Limitation Score.

^a^ Negative correlation.

In the secondary analysis with the two electrophysiological parameters, the CT‐RC correlated inversely with ƩCMAP (*p* = 0.006) and ƩSNAP (*p* = 0.027).

### Independent predictors of total ONLS improvement

Linear regression results are summarized in Table 4. Pretreatment total ONLS (*p* < 0.001) and acuteness of presentation (*p* = 0.015) independently predicted amplitude of ONLS improvement.

**TABLE 4:** Significance level results of linear regression analyses for independent predictors of CT‐RC and total ONLS improvement among relevant covariables in the total cohort of 112 consecutive subjects with treated CIDP.

	Age	Disease duration	Typical CIDP subtype	Acuteness of presentation	Comorbidity	Pretreatment total ONLS
CT‐RC	*p* = 0.035	NS	NA	NA	NS	NA
Total ONLS improvement	NA	NS	NA	*p* = 0.015	NA	*p* < 0.001

Abbreviations: CIDP, chronic inflammatory demyelinating polyneuropathy; CT‐RC, CIDP treatment‐response category; NA, nonapplicable; NS, nonsignificant; ONLS, Overall Neuropathy Limitation Score.

Of the two electrophysiological parameters studied, only ƩCMAP independently predicted the CT‐RC (*p* = 0.049).

### Subanalysis of treatment‐naïve subjects

Considering exclusively the 70 treatment‐naïve subjects in our cohort, the CT‐RC correlated only inversely with ONLS improvement (*p* < 0.001) but not with age (*p* = 0.015, Bonferroni‐corrected significance *p* < 0.007). Time to maximum improvement did not correlate with any pretreatment covariate. Treatment discontinuation at the time of study correlated only inversely with disease duration before treatment initiation (*p* < 0.001). The amplitude of ONLS improvement correlated inversely with disease duration before treatment initiation (*p* < 0.001), directly with acuteness of presentation (*p* < 0.002), and with pretreatment total ONLS (*p* < 0.001). Only pretreatment total ONLS (*p* < 0.001) independently predicted amplitude of ONLS improvement.

## DISCUSSION

Outcome and its timing are uncertain in subjects with CIDP receiving routine care. Using the exclusively result‐based CT‐RC, we found that one third of treated patients regained complete function and another one third a good but partial level of function. Of the remaining one third, two thirds had a moderate or poor response and one third was nonresponsive. These findings indicate suboptimal outcomes with current treatments and/or treatment methods in a majority of subjects with CIDP. Of note, the CT‐RC was only independently predicted by age, challenging prognostication based on other parameters.

The mean timing of the maximum improvement achieved during the 36‐month evaluation period, although highly variable, was approximately 1 year. A gradually incremental rate to maximum improvement was observed from 20% at 3 months to 60% at 12 months, with 40% of subjects only reaching their maximum improvement between 12 and 36 months. Delayed responses have been documented in CIDP with corticosteroids [14] and intravenous immunoglobulins [18], although the current findings are not comparable, as they include treatment modifications performed along the way and relate to the maximum improvement achieved instead of a prespecified MID‐defined response. These findings highlight the potential for continuing amelioration long after treatment initiation. Importantly, time to maximum improvement could not be predicted by any pretreatment variable, suggesting that treatment perseverance/escalation may be justified in all partial responders in an attempt to further improve outcomes, particularly in the absence of plateauing of patient proportions reaching maximum improvement in our cohort during the 36‐month follow‐up period.

We found that treatment cessation was more frequent in subjects with a shorter pretreatment disease duration, which was also associated with larger ONLS improvement. It has previously been shown that axonal loss, as occurs with delayed treatment, results in treatment unresponsiveness in CIDP [19]. Our findings, in addition, suggest that delayed treatment may also be more likely to result in smaller amplitude amelioration as well as prolonged, treatment‐requiring, active disease.

The mean number of treatments used was comparable in responders and nonresponders, suggesting possible insufficient escalation in the latter group, in view of the similar disease severity in the two groups. The greater age of nonresponders may partly explain this. However, it may conversely also be possible that response is poorer in CIDP in older age, despite additional therapies.

Treatment discontinuation in partial responders, commonly performed and observed in our cohort, may be questionable practice, in view of the delayed time to maximum improvement. Such discontinuation implies physician and/or patient satisfaction with the suboptimal functional level reached. Similarly, absence of full escalation to all available therapeutic options in nonresponders suggests lack of physician and/or patient willingness or ability to do so. As such, physician‐ and patient‐related factors appear clearly implicated in routine care and its outcomes in CIDP. Anchoring bias [20, 21, 22], defined as overreliance on initial information (here, improvement amplitude, perceived prognosis from previous experience, or perceived long‐term treatment requirements), with lack of consideration for other relevant evidence (here, potential for further improvement, possible delayed treatment effects, or appropriateness of escalation), is likely relevant in CIDP. Therapeutic inertia (TI), defined as absence of treatment initiation or intensification when treatment goals are unmet [23, 24, 25, 26], may also be implicated. With relevance to CIDP, TI has been reported as affecting management of older subjects more commonly [27]. TI has been shown to impact management of multiple sclerosis [28, 29], and although not studied to date, it is probable that it may also be implicated in CIDP, particularly in the absence of a reliable biomarker of disease activity. Similarly, clinical experience in CIDP and existing literature in other diseases suggest direct patient‐related factors, due to low expectations [30], dissatisfaction with treatment inconvenience [31], and experienced or feared side effects [32], may also have impacted upon therapeutic management of partial responders and non‐responders.

With regard to the electrophysiological parameters considered, the pre‐treatment degree of motor axonal loss independently predicted CT‐RC, in line with the findings of previous studies [19, 33].

Our study has a number of limitations, including its retrospective single‐centre design and the number of subjects included. We used for consistency a single outcome measure and did not consider ONLS nonresponders, who showed changes on other scales such as the I‐RODS. Dissimilar score evolution and interscale discrepancy [34, 35], as well as patient‐perceived I‐RODS item inadequacy [36], were all dissuading factors to include additional outcome measures in CT‐RC generation. However, 10 of 14 non‐responders demonstrated MID‐defined response on other outcome measures, leading to continuing treatment. Further work is needed to evaluate the clinical relevance of the benefit and factors involved in this subgroup. We determined the timing of maximum improvement documented at assessment at clinical review, also considering recent patient‐reported history. This may have caused underestimation, but, importantly, not overestimation, of time to maximum improvement. Finally, our cohort included more than one third of subjects referred from other institutions and with long disease durations. This may have impacted upon the findings, as the studied population may have comprised a larger than average proportion of poor responders to treatment. It is nonetheless likely that our cohort was comparable with those attending most tertiary centres. Of note, subanalysis performed on treatment‐naïve patients yielded comparable results to those of the full cohort, except for lack of association between CT‐RC and age, and of an independent association of ONLS improvement with acuteness of presentation. In the former case, increased type II error, due to Bonferroni correction, of debated application in exploratory studies such as ours [37, 38], and in the latter, the smaller sample size, may explain these differences.

We believe our study shows novel findings on response characterization, rates of persistent disability, and delayed response timing in CIDP with routine care. The proposed CT‐RC shows high concurrent validity with other existing outcome measures, while additionally providing classification into functionally meaningful response groups. It furthermore enables accurate and relevant quantification of the global residual unmet therapeutic need with routine care. The findings on timing of maximum improvement may question current practice of treatment reduction and/or withdrawal in partial responders and require further research. Finally, the results, including the limited escalation in non‐responders, offer insight into the impact of physician‐ and patient‐related factors on outcomes, which equally merit further study.

## AUTHOR CONTRIBUTIONS

**Yusuf A. Rajabally:** Conceptualization; writing – original draft; methodology; formal analysis; project administration; supervision. **Young Gi Min:** Formal analysis; writing – original draft; methodology. **Kabir K. Nazeer:** Writing – review and editing; formal analysis. **Christina Englezou:** Formal analysis; writing – review and editing.

## CONFLICT OF INTEREST STATEMENT

Y.A.R. has received consultancy honoraria from Sanofi, Janssen, Argenx, LFB, Polyneuron, Takeda, and Dianthus; has received educational sponsorships from LFB and CSL Behring; and has obtained research grants from LFB. K.K.N. has obtained a research grant from LFB. Y.G.M. and C.E. have no disclosures.

### ETHICS APPROVAL

This study was part of a retrospective clinical audit approved and registered by the University Hospital Birmingham NHS Foundation Trust Clinical Audit Service (CARMS no. 20702, 23 October 2023). Clinical audits do not require ethics committee approval in the UK.

## Data Availability

The data that support the findings of this study are available on request from the corresponding author. The data are not publicly available due to privacy or ethical restrictions.
